# Gestation Related Gene Expression of the Endocannabinoid Pathway in Rat Placenta

**DOI:** 10.1155/2015/850471

**Published:** 2015-07-02

**Authors:** Kanchan Vaswani, Hsiu-Wen Chan, Hassendrini N. Peiris, Marloes Dekker Nitert, Ryan J. Wood Bradley, James A. Armitage, Gregory E. Rice, Murray D. Mitchell

**Affiliations:** ^1^University of Queensland Centre for Clinical Research, Royal Brisbane and Women's Hospital Campus, Herston, QLD 4029, Australia; ^2^Department of Anatomy & Developmental Biology, Monash University, Clayton, VIC 3800, Australia; ^3^School of Medicine (Optometry), Deakin University, Pigdons Road, Waurn Ponds, VIC 3800, Australia

## Abstract

Mammalian placentation is a vital facet of the development of a healthy and viable offspring. Throughout gestation the placenta changes to accommodate, provide for, and meet the demands of a growing fetus. Gestational gene expression is a crucial part of placenta development. The endocannabinoid pathway is activated in the placenta and decidual tissues throughout pregnancy and aberrant endocannabinoid signaling during the period of placental development has been associated with pregnancy disorders. In this study, the gene expression of eight endocannabinoid system enzymes was investigated throughout gestation. Rat placentae were obtained at E14.25, E15.25, E17.25, and E20, RNA was extracted, and microarray was performed. Gene expression of enzymes *Faah, Mgll, Plcd4, Pld1, Nat1, Daglα*, and *Ptgs2* was studied (cohort 1, microarray). Biological replication of the results was performed by qPCR (cohort 2). Four genes showed differential expression (*Mgll, Plcd4, Ptgs2, and Pld1*), from mid to late gestation. Genes positively associated with gestational age were *Ptgs2, Mgll, and Pld1*, while *Plcd4* was downregulated. This is the first comprehensive study that has investigated endocannabinoid pathway gene expression during rat pregnancy. This study provides the framework for future studies that investigate the role of endocannabinoid system during pregnancy.

## 1. Introduction

During gestation, products of the endocannabinoid system in the placenta are crucial for maintenance of pregnancy and for both the onset and progression of labor [1]. The endogenous ligands for this system, the endocannabinoids, are formed from membrane phospholipids. Two well characterised endocannabinoids are 2-acylglycerol (2AG) and anandamide (AEA). These molecules are not stored within cells but are synthesised and released in response to increased substrate availability and synthase activity [2]. 2AG and AEA bind to the G-coupled protein receptors, cannabinoid receptors 1 and 2 (CB1 and CB2) [1]. The loss of the CB receptors has been linked to preterm delivery which accounts for 5–18% of pregnancies worldwide [3, 4]. Thus this complex endocannabinoid system may play a role in preterm birth [5].

**Figure 1:**
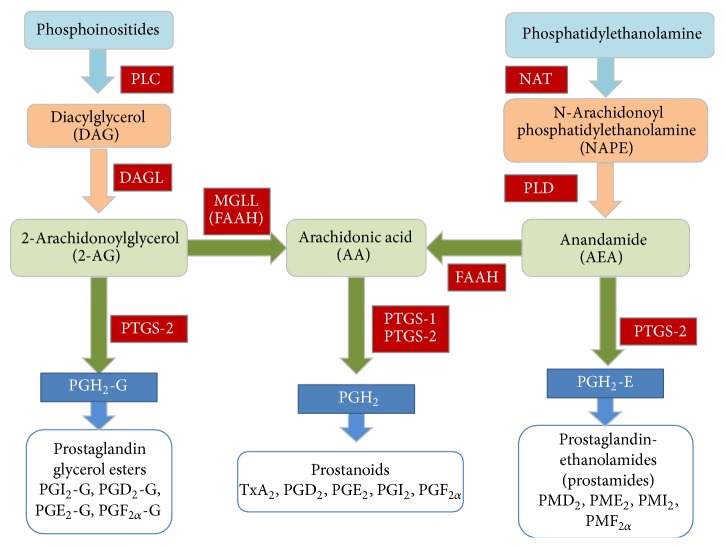
Endocannabinoid system giving rise to endocannabinoids (2AG and AEA) and arachidonic acid and subsequently synthesis of prostaglandin glycerol esters, prostanoids, and prostamides.

The endocannabinoid synthesis system is complex (Figure 1). AEA is believed to be formed by a two-step catalysis in which arachidonic acid is transferred to a phospholipid precursor, phosphatidylethanolamine, by N-acyltransferases (NATs) to form N-arachidonoyl phosphatidylethanolamine (NAPE). NAPE is cleaved by phospholipase D (PLD) to form AEA [6]. 2AG is synthesised from diacylglycerol through the action of diacylglycerol lipase (DAGL) [7]. Diacylglycerol is formed from phosphoinositides by the action of an important enzyme, phospholipase C (PLC). The liberation of arachidonic acid occurs via the catalysis of enzyme phospholipase A2 or indirectly by the catalyses of PLC and DAGL and monoacyl glycerol lipase enzymes (MAGL, protein coded by* Mgll*) [8]. FAAH converts AEA to arachidonic acid and ethanolamine (Figure 1).

Arachidonic acid can be converted to PGH2 by the enzymes PTGS-2 and PTGS-1. PGH2 is further converted to other key downstream molecules/metabolites of the eicosanoid family (prostaglandin glycerol esters, prostanoids, and prostamides) that have been studied for their instrumental roles in the aetiology of labor, namely, TxA2, PGD2, PGE2, PGI2 and PGF2a, and so forth (Figure 1). These are synthesised by the action of other downstream enzymes. PTGS2 is crucial for its multifactorial role, as it also converts anandamide to PGH2-E and 2AG to PGH2-G [9]. In rat placenta, 2AG and AEA play important roles in fetoplacental development and aberrant cannabinoid signalling can cause pregnancy complications [10, 11]. PGF2*α* is particularly important for labor since it induces myometrial contractility [12–14].

This paper focuses on the endocannabinoid system from mid to late gestation leading up to the period just prior to labor. Distorted expression of genes and their metabolites of the endocannabinoid system can result in spontaneous miscarriage and poor pregnancy outcomes such as preterm birth. Recently it has been suggested that the endocannabinoid profiles can serve as biomarkers in a clinical setting to identify and/or predict spontaneous preterm labor [15]. To date, limited studies have reported on placental gene expression of these enzymes from mid pregnancy to labor. The aim of this study is to compare the expression changes of eight enzymes involved in the endocannabinoid system from rat placenta taken at 4 gestational stages E14.25, E15.25, E17.25, and E20. Placentae from two independent cohorts were used in this study. The hypothesis of this study was that endocannabinoid enzyme expression would change (either increase or decrease) throughout gestation, in support of a normal gestation. Although placental factors cannot be used directly as biomarker(s) to predict and/or identify spontaneous labor onset in a clinical setting, as placental biopsies will not be available prior to labor, the study may be able to provide insight into what factors need further investigation in biological fluids that can be quantified during pregnancy.

## 2. Methods and Materials

### 2.1. Study Design

**Figure 2:**
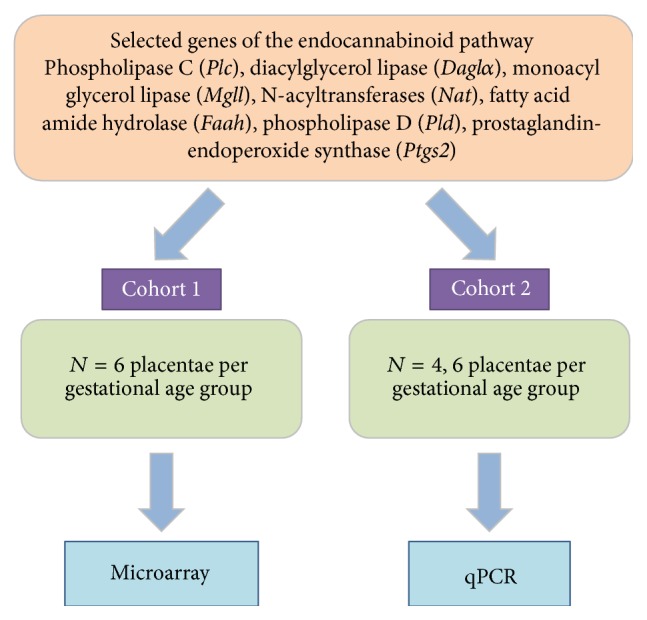
Study design: two independent cohorts of rat placenta samples used for microarray (cohort 1) and qPCR (cohort 2). In cohort 2, we used E14.25, *n* = 6, E15.25, *n* = 4, E17.25, *n* = 6, and E20, *n* = 6. Note:* Ptgs1* was only analyzed by qPCR.

Genes were selected based on their involvement in the endocannabinoid system (as shown in Figure 1), specifically genes for enzymes that are involved in the synthesis of the molecules PGH2, AEA, and 2AG and arachidonic acid, namely,* Plc, Daglα, Mgll, Nat, Faah, Pld, Ptgs1*, and* Ptgs2*. The expression of these genes, except* Ptgs1*, was analyzed using microarray on a first cohort of rat placental samples. A second cohort of independent samples was then used for biological replication as validation by qPCR as shown in Figure 2.

### 2.2. Animals and Diets

All animal experiments were performed with the approval of The School of Biomedical Sciences' Animal Ethics Committee of Monash University. Experiments were carried out in accordance with the National Health and Medical Research Council of Australia* “Australian Code of Practice for the Care and Use of Animals for Scientific Purposes”* (7th edition, 2004).* Sprague Dawley* rats were used throughout this study. Healthy dams were allowed to adapt to the animal house for one week. They were fed a standard chow diet (20% protein, 68% carbohydrate, and 12% fat) and water* ad libitum* in a light controlled environment (12 h light/dark cycle) throughout the study. Female rats were time-mated for 3 h with male* Sprague Dawley* rats, to reduce variability of gestational age among the offspring and to maximize the accuracy in staging of gestation. Dams were individually housed after mating. Both cohort samples (cohorts 1 and 2) were fed and treated in the same fashion to maintain consistency.

### 2.3. Tissue Collection

Pregnant dams were anaesthetized (Isoflurane Rhodia Australia P/L, VIC, Australia) and humanely killed at embryonic days (E) 14.25, 15.25, 17.25, and 20 (*n* = 6 per gestational age). Whole placentae were collected from each of the pregnant dams, weighed, and snap-frozen in liquid nitrogen. Tissues were stored at −80°C until being processed and analyzed.

### 2.4. RNA Isolation

Using a mortar and pestle, rat placental tissues were pulverized into a very fine powder using liquid nitrogen. Total RNA was extracted from 30 mg of pulverized frozen placental tissue using the AllPrep DNA/RNA Mini Kit (Qiagen) as per manufacturers' instructions. Genomic DNA removal was ensured by an on-column Dnase1 treatment step. Total RNA was quantified via NanoDrop ND-1000 spectrophotometer (Thermo Scientific, DE, USA). RNA integrity was verified using an Agilent 2100 Bioanalyzer (VIC, Australia), by RIN (RNA Integrity Number) score measurements prior to the analysis. RNA samples that fulfilled the following criteria were selected for microarray analysis: (i) RIN > 8.5; (ii) 260/280 ratio > 2; (iii) 260 : 230 > 1. All RIN scores were greater than 8.7.

### 2.5. Microarray Analysis on Illumina Rat Ref Arrays

For the microarray analysis, 500 ng of total RNA was converted to double stranded cDNA and this was used to generate biotinylated cRNA probes using the Illumina TotalPrep RNA Amplification Kit. Biotin-labelled cRNA were then hybridized to Illumina RatRef-12 Expression BeadChip (San Diego, CA, USA). Slides were scanned on a BeadStation 500 System using Beadscan software Version 3.5.31. Each of the placental samples was analysed independently. Array experiment readout was deposited on ArrayExpress (ArrayExpress accession number E-MTAB-1987). Overall microarray results were previously published by our group [16]. For this study, expression of the 7 enzymes* Faah, Mgll, Plcd4, Pld1, Nat1, Daglα,* and* Ptgs2* in the endocannabinoid system was analyzed.

### 2.6. Quantitative Real-Time PCR Validation Experiments

qPCR was used to confirm and validate the expression of 8 genes,* Faah, Mgll, Plcd4, Pld1, Nat1, Daglα, Ptgs1* (data not shown, not carried out in microarray), and* Ptgs2* in an independent cohort on an ABI StepOne PCR machine. All primers were designed specifically for rat sequences only spanning intron junctions, to eliminate amplification of genomic DNA. The primer sequences are listed in Table 1. Supplementary Figure S2 (see Supplementary Material available online at http://dx.doi.org/10.1155/2015/850471) displays the amplification products of the various genes using these primers displayed in Table 1. *β*-actin was used as the endogenous control gene and relative expression was calculated using the ΔΔCT method.

## 3. Results

### 3.1. Gestational Age-Related Expression Changes of 4 Endocannabinoid System Enzymes

**Figure 3:**
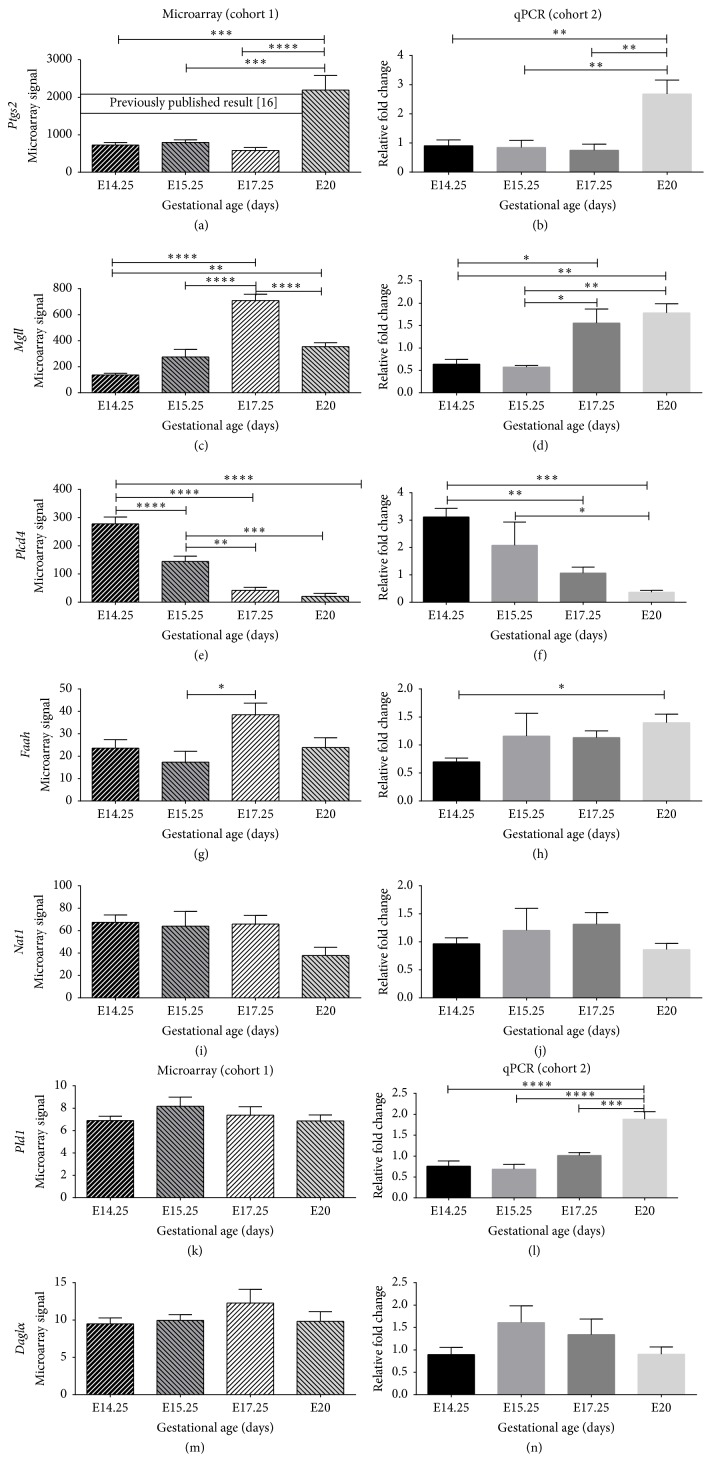
Differential expression of seven eicosanoid enzyme genes assessed by microarray (cohort 1) and validated by qPCR (cohort 2). The left panel shows the results of the microarray analyses; the right panel displays the results of the qPCR. (a)* Ptgs2* by microarray in cohort 1 has been previously published. The level of statistical significance is shown as asterisk. Gene expression changes were considered significant when gene expression was changed by >2 (^*∗*^ *P* < 0.05; ^*∗∗*^ *P* < 0.005; ^*∗∗∗*^ *P* < 0.001; ^*∗∗∗∗*^ *P* < 0.0001; and ^ns^ *P* > 0.05).

**Figure 4:**
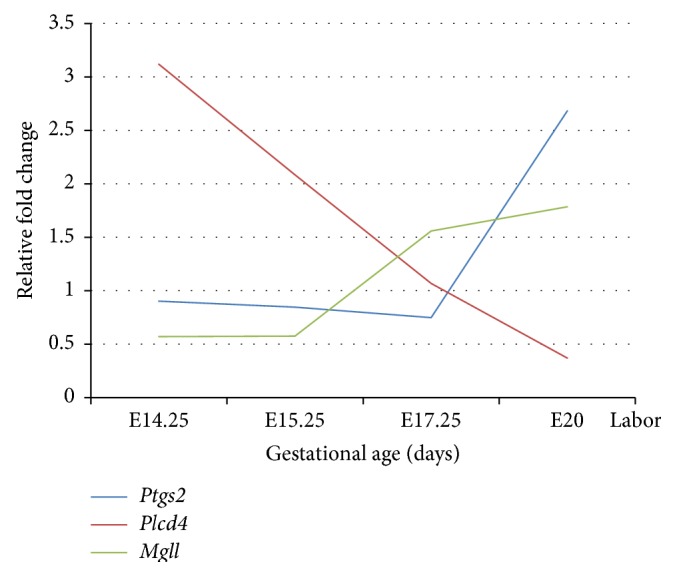
Proposed relationship of the 3 differentially expressed genes* Ptgs2, Plcd4,* and* Mgll *from mid to late gestation from qPCR results.

Of the 7 endocannabinoid enzymes involved in the metabolism of endocannabinoids, 4 (*Ptgs2, Mgll, Plcd4*, and* Pld1*) were differentially expressed in cohort 2 samples by qPCR. Cohort 1 samples showed differential expression of* Ptgs2, Mgll,* and* Plcd4* but not* Pld1* (Figure 3).* Ptgs2* showed a marked increase in expression between E17.25 and E20 of fold change of >2.5 (*P* < 0.001),* Mgll* showed consistent 3-fold increase from E15.25 to E17.25 (*P* < 0.05; qPCR and <0.001; microarray), and* Plcd4* showed a gradual decrease in expression from E14.25 to E15.25 (fold change approximately 2, *P* < 0.05) and E17.25 with expression remaining relatively low at E20 just prior to labor onset. A gene expression pattern for these 3 genes (*Ptgs2, Mgll, and Plcd4*) is shown in Figure 4.

### 3.2. Gene Expression That Remained Unchanged from Mid to Late Gestation

The gene expressions of 8 enzyme genes were carried out, out of which* Nat1, Daglα*, and* Faah* expression remained relatively stable throughout gestation in both cohorts 1 (microarray) and 2 (qPCR) as shown in Figure 3.* Ptgs1 (data not shown)* was only studied by qPCR and showed no differential expression (constitutive expression), consistent with literature.

### 3.3. Other Endocannabinoid Genes Whose Expression Remained Relatively Stable in Microarray

Interestingly the expression of other genes in the system that were stable over the course of gestation included the phospholipases* Plcb3, Plcd3, Plcl3, Plcz1, Pld2* and* Pld3*, and* Nat2* and* Nat3*; the genes for phospholipase 2 isotypes* Pla2g15, Pla2g2d, Pla2g2e, Pla2g3*, and* Pla2g5*; and genes for cannabinoid receptors CB1 and CB2, that is,* Cnr1* and* Cnr2* (data not shown).

## 4. Discussion

This study is the first to highlight differential expression of 4 genes (*Plcd4, Ptgs2, Pld1,* and* Mgll*) in rat placenta from mid-late gestation to labor onset. Our study expands on a previous study [11] in rat placenta that has focused on expression of CB1 and CB2 endocannabinoid receptor, genes (Cnr1 and Cnr2), and some genes of the endocannabinoid system. Most other studies of placenta from normal and pathological pregnancies have focused on the downstream prostaglandins (particularly PGH2) as an end product, some with the involvement of* Ptgs1 and Ptgs2*, during the onset of labor [17]. Consistent with previous literature* Daglα* and* Faah* expression [11] and* Ptgs1 and Nat1* expression remained stable throughout the four gestational ages.

### 4.1. *Ptgs2* and* Ptgs1* in This Study and in Pregnancy

*Ptgs1* and* Ptgs2* have been widely studied in placentae of women and animals models for decades [18]. Here we observe that expression of* Ptgs2* in whole rat placenta in both cohorts remained stable throughout E14.25, E15.25, and E17.25 but was significantly upregulated at E20, just before the onset of labor, consistent with previous studies [16, 19–21]. Since* Ptgs1* and* Ptgs2* are directly involved in prostaglandin syntheses, any differential or aberrant expression of these two enzymes may have a dramatic effect in prostaglandin levels [19]. We also know that* Ptgs2* is an inducible gene, which may be triggered to initiate the labor process. However it cannot be determined conclusively whether the increase of* Ptgs2* leads to prostaglandin production, which then triggers the labor process (which is the ongoing hypothesis in various animal studies). In fact, some studies have suggested that PGs originating in the fetal membranes are a trigger for the onset of normal labor [21]. This sudden observed spike in* Ptgs2* expression is associated with increased production of prostaglandins. Increased prostaglandins are observed just prior to and during labor. Further studies on* Ptgs2* in mammalian pregnancy and labor onset are required given its variable expression and vital role in the biosynthesis of prostaglandins.* Ptgs1* on the other hand is a constitutively expressed gene and by qPCR demonstrated that its expression remained relatively stable throughout mid to late gestation (data not shown).* Ptgs2* is important in the endocannabinoid system and there is now evidence to show that factors (PGE2,* Ptgs2,* etc.) involved in this pathway in placenta may have a role in ovulation, implantation, and decidualization [22, 23] and abnormal expression may result in spontaneous miscarriage, placental abruptions, and poor pregnancy outcomes [24, 25]. Prostaglandins increase myometrial sensitivity and PTGS2 is especially associated with changing myometrial contractility during pregnancy [26]. In a clinical setting, selective PTGS2 inhibition is used to delay premature onset of labor [13]. Although nonsteroidal anti-inflammatory drugs (NSAIDs), which inhibit PTGS2, are usually successful in suppressing preterm labor or prolonging pregnancy in animal and human studies, the NSAIDs have adverse effects on fetal physiology and development, including renal defects and cardiomyopathies [5, 26, 27]. The need then arises for another approach to predict and treat preterm labor. Fluctuations in gene expression and enzyme concentrations and metabolite production may serve as useful indicators of labor onset. These factors, their metabolites, and substrates may then be further investigated in biological fluids to study their potential as biomarkers for early detection of preterm birth and hence pregnancies where there is a risk of preterm delivery to be captured early.

*Ptgs2* regulation occurs at many levels [28] and is affected by transcription factors, activator proteins, and chromatin remodelling. Moreover, the endocannabinoid system has come under scrutiny given that several microRNAs such as miR26, miR146, and miR10 are responsible for its gene regulation [28, 29]. These have emerged as regulators of not only physiological processes of pregnancy and labor, such as uterine contractions [30–32]. A future study to link gene regulation patterns and labor is necessary. Also, other prostaglandin and thromboxane molecules should be investigated further to determine their roles in pregnancy and the inflammatory process.

### 4.2. Phospholipase C (PLC)

Interestingly, another novel gene that showed significant differential expression was* Plcd4*.* Plcd4* expression was relatively high at E14.25 and gradually decreased at E15.25, E17.25 and further decreased at E20.* Plcd4* expression was significantly reduced just prior to labor onset in both cohorts. Arachidonic acid can be synthesized through the conversion of phosphoinositides to diacylglycerol by PLC. The results perhaps indicate that the product of this enzyme, diacylglycerol, is reduced and that PLCD4 may be necessary to control levels of diacylglycerol and subsequently 2AG and PGH2-G. This control mechanism could be an important player in the process of labor and hence this study should be further expanded in preterm samples as there is a paucity of data with regard to the role of* Plcd4* in pregnancy. Apart from identifying the presence of the gene transcripts in myometrium from pregnant versus nonpregnant rats,* Plcd4*, unlike phospholipase A [16, 33], has not been studied for its role in placenta especially with its link to labor and parturition. Nevertheless PLCD4 can be further investigated in human samples as a potential biomarker for preterm deliveries in perhaps other gestational tissues, including membranes and/or biological fluids, and a reduced level of the enzyme could be a useful prognostic for impending preterm birth.

Another gene to be highlighted in this study for its marked gene expression change from E15.25 to E17.25 is* Mgll* where a 2-fold increase was observed in both cohorts (E15.25 to E17.25). This increase likely indicates that downstream synthesis of arachidonic acid may be present. An increase of arachidonic acid at E17.25 and then a subsequent increase in expression of* Ptgs2* at E20 are likely to increase arachidonic acid derived PGH2. Interestingly, when* Plcd4* expression is at its lowest level, then* Ptgs2* expression is at its highest level, just prior to labor. A relationship between the 3 genes* Ptgs2, Mgll,* and* Plcd4* and time points of gestation is shown in Figure 4.

### 4.3. Other Endocannabinoid System Genes

From qPCR results on cohort 2 samples,* Pld1* expression increased steadily from E14.25, E15.25, and E17.25 to E20. Interestingly microarray signals were below the level to detect change as it has a very low expression level overall. Steady expression of* Faah* across gestation observed in the present study is consistent with the findings of Mijovic et al. in rat placenta [15].

In both qPCR and microarray, the expression profile of* Daglα is* consistent with the literature [15].* Daglα* and* Nat1* expression were both observed to remain stable in mid to late gestation, indicating their constitutive expression during this time window likely maintaining the function of their by-products of metabolism NAPE (precursor for anandamide) and 2AG, respectively, throughout pregnancy. Interestingly the expressions of other enzyme genes, in microarray, in the system that did not change during the gestation window are* Nat3, Plcb3, Plcd3, Plcl3, Plcz1, Pld2, Pld3, Pla2g15, Pla2g2d, Pla2g2e, Pla2g3, and Pla2g5* (data not shown). Other related genes from microarray data are shown in Supplementary Figure S1.

**Figure 5:**
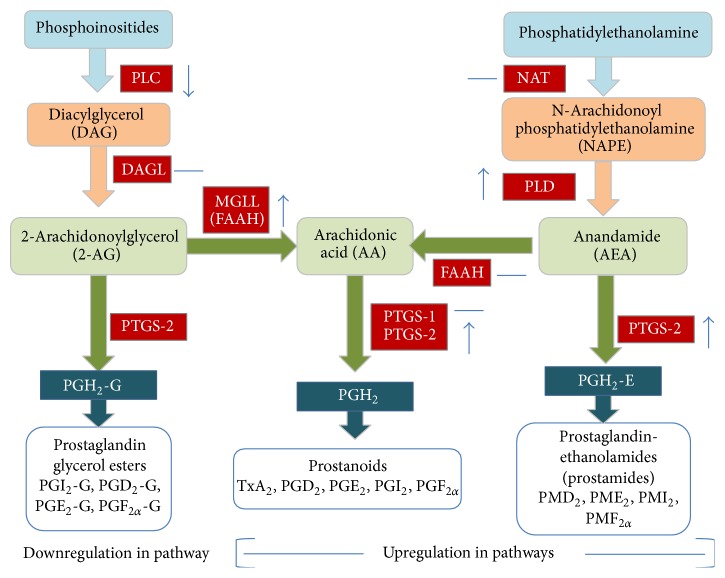
Proposed model of gestational related changes in gene expression of endocannabinoid system genes, as pregnancy progresses from mid to late gestation in rat placenta.

From the results of this study, summarized in Figure 5, increasing gestational age is associated with increasing synthesis of PGH-2 (and downstream metabolites, prostanoids, and prostamides) from AA and AEA, respectively (Figure 5). On the other hand, the formation of prostaglandin glycerol esters may be downregulated as gestation progresses. Both AEA and AA are metabolites of PLD and MAGL, respectively, and genes for these two enzymes show upregulation from mid to late gestation. This is complemented by the study by Fonseca et al., 2012, which shows that AEA levels in the placenta gradually increased reaching maximum level on day 19 of gestation, while expression remained lower on days 14 and 16 [11]. The downregulation in* Plcd4* may indicate a control mechanism that reduces the synthesis of 2AG and hence PGH2-G and its derivatives. There is a paucity of data pertaining to the formation of glycerol esters in late stage pregnancy and labor. This shift in gene regulation away from 2AG metabolism may be key in studying and choosing the metabolites for prediction of preterm birth. Furthermore, it is predicted that increases in arachidonic acid concentration would arise by 2AG and AEA metabolism, prior to PGH2 formation by PTGS2. The data provides an opportunity to further elucidate functional pathways that may be of significance in late placental function in both normal and pathological pregnancies.

## 5. Conclusion

From the results of this study, late gestation is associated with the synthesis of PGH-2 (and their downstream metabolites, prostanoids, and prostamides) from AA and AEA, respectively. The formation of prostaglandin glycerol esters may be downregulated as gestation progresses. In this study, we show that the genes for PLD and MAGL, which catalyse the synthesis of both AEA and AA, are upregulated from mid to late gestation, while the* Plcd4* expression is downregulated. This may indicate a regulatory mechanism that reduces the synthesis of 2AG and hence PGH2-G and its derivatives. This shift in gene regulation away from 2AG metabolism could be a key pathway for the identification of metabolites for the prediction of preterm birth.

## Supplementary Material

Supplementary Data includes the differential expression of other genes related to the endocannabinoid system that were screened in Microarray experiments and also the amplification products of Primers used in the study.

## Figures and Tables

**Table 1:** Primer sequences (forward and reverse) and product lengths for all genes used in qPCR experiments.

Gene	Product	Forward primer	Reverse primer
*Ptgs2 *	201 bp	TCACCCGAGGACTGGGCCAT	CAGCGAACCGCAGGTGCTCA
*Mgll *	240 bp	CGCGCAGTAGTCTGGCTCT	AAGATGAGGGCCTTGGGTGTG
*Plcd4 *	182 bp	CTTCCAGTCGTCAGACTACCC	CCAAGATCTTCCCCCGAAGC
*Faah *	218 bp	CAACCGCCTCAGCAAGAGTG	CATAGTACCCCACACGCAGG
*Dagla *	121 bp	CTTGGACTCAGCCCTGGAC	GAACTGGGATGAAGGGGGTG
*Nat1 *	240 bp	TCACTCGCCATGGCATTCTC	AGCCTGGCTTGCTTTTACCT
*Pld1 *	224 bp	CCCCCATCTCGACTTTCAACT	TCCCGGGTGTCGAGATTTTC
*Ptgs1 *	145 bp	TAGGCCATGGGGTAGACCTT	TTCACGGACGCCTGTTCTAC
*β-actin *	119 bp	TCCACCCGCGAGTACAACCT	TTGCACATGCCGGAGCCGTT
