# Supplementary material for: Gestation Related Gene Expression of the Endocannabinoid Pathway in Rat Placenta
Source: Mediators Inflamm. 2015 Jul 2;2015:850471. doi: 10.1155/2015/850471 (PMC4503552; doi:10.1155/2015/850471)
Supplement: Supplementary file 1 — Supplementary Data includes the differential expression of other genes related to the endocannabinoid system that were screened in Microarray experiments and also the amplification products of Primers used in the study. [file 850471.f1.docx]

**A**


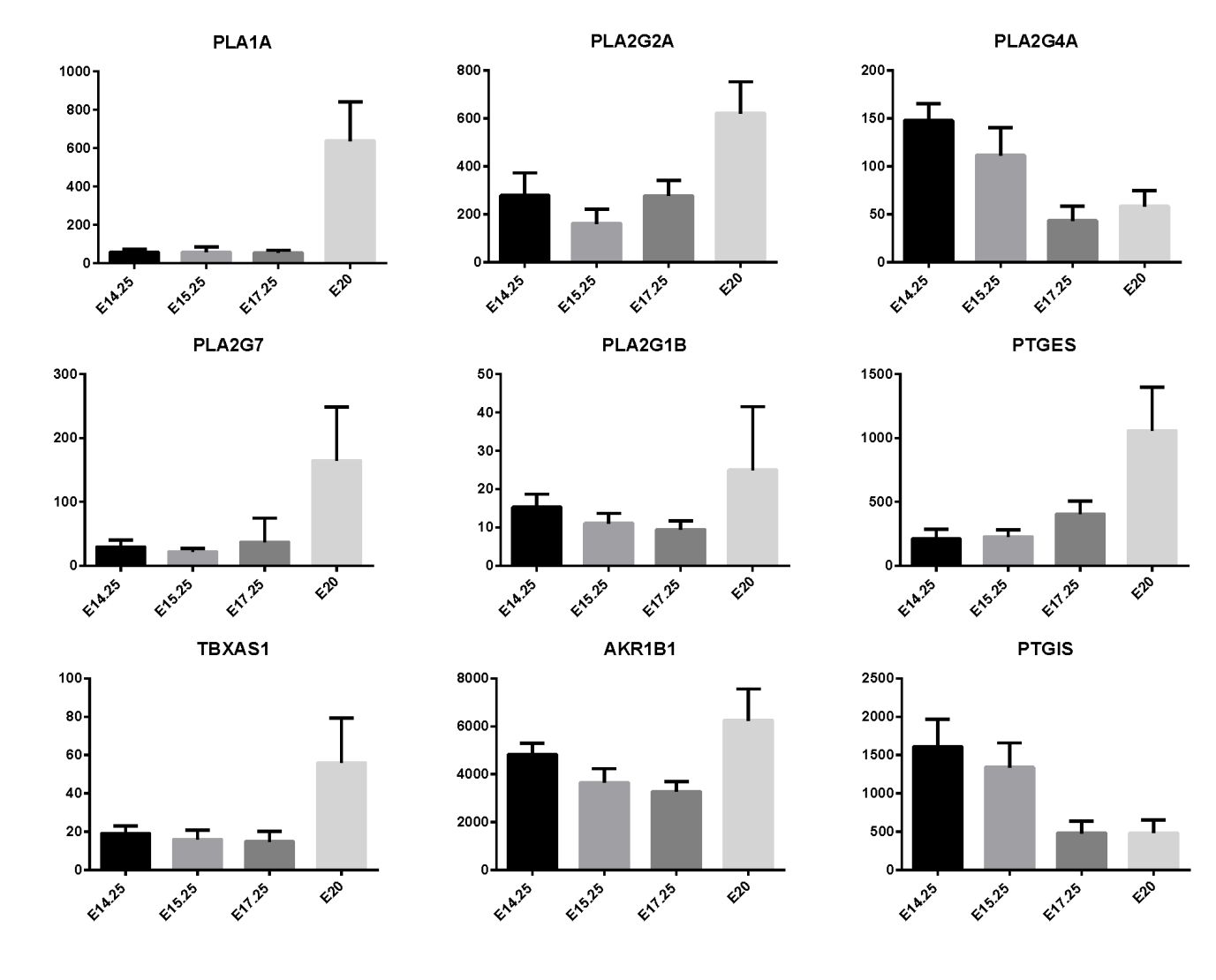
**B**

| *Tukey's multiple comparisons test :Pla1a* | *Mean Diff.* | | *95% CI of diff.* | | | *Significant?* | | | *Summary* | | |  |
| --- | --- | --- | --- | --- | --- | --- | --- | --- | --- | --- | --- | --- |
|  |  | |  | | |  | | |  | | |  |
| *E14.25 vs. E15.25* | *-1.761* | | *-168.4 to 164.9* | | | *No* | | | *ns* | | |  |
| *E14.25 vs. E17.25* | *1.148* | | *-165.5 to 167.8* | | | *No* | | | *ns* | | |  |
| *E14.25 vs. E20* | *-581.8* | | *-748.5 to -415.1* | | | *Yes* | | | ****** | | |  |
| *E15.25 vs. E17.25* | *2.909* | | *-163.8 to 169.6* | | | *No* | | | *ns* | | |  |
| *E15.25 vs. E20* | *-580.0* | | *-746.7 to -413.4* | | | *Yes* | | | ****** | | |  |
| *E17.25 vs. E20* | *-582.9* | | *-749.6 to -416.3* | | | *Yes* | | | ****** | | |  |
|  |  | |  | | |  | | |  | | |  |
| *Tukey's multiple comparisons test :Pla2g2a* | *Mean Diff.* | | *95% CI of diff.* | | | *Significant?* | | | *Summary* | | |  |
|  |  | |  | | |  | | |  | | |  |
| *E14.25 vs. E15.25* | *117.3* | | *-31.25 to 265.9* | | | *No* | | | *ns* | | |  |
| *E14.25 vs. E17.25* | *0.6701* | | *-147.9 to 149.3* | | | *No* | | | *ns* | | |  |
| *E14.25 vs. E20* | *-343.6* | | *-492.1 to -195.0* | | | *Yes* | | | ****** | | |  |
| *E15.25 vs. E17.25* | *-116.7* | | *-265.3 to 31.92* | | | *No* | | | *ns* | | |  |
| *E15.25 vs. E20* | *-460.9* | | *-609.5 to -312.3* | | | *Yes* | | | ****** | | |  |
| *E17.25 vs. E20* | *-344.2* | | *-492.8 to -195.6* | | | *Yes* | | | ****** | | |  |
| *Tukey's multiple comparisons test :Pla2g4a* | | *Mean Diff.* | | *95% CI of diff.* | *Significant?* | | | *Summary* | | |  |  |
|  | |  | |  |  | | |  | | |  |  |
| *E14.25 vs. E15.25* | | *36.35* | | *3.597 to 69.10* | *Yes* | | | *** | | |  |  |
| *E14.25 vs. E17.25* | | *104.4* | | *71.63 to 137.1* | *Yes* | | | ****** | | |  |  |
| *E14.25 vs. E20* | | *89.31* | | *56.56 to 122.1* | *Yes* | | | ****** | | |  |  |
| *E15.25 vs. E17.25* | | *68.03* | | *35.28 to 100.8* | *Yes* | | | ****** | | |  |  |
| *E15.25 vs. E20* | | *52.96* | | *20.21 to 85.71* | *Yes* | | | **** | | |  |  |
| *E17.25 vs. E20* | | *-15.07* | | *-47.82 to 17.68* | *No* | | | *ns* | | |  |  |
| *Tukey's multiple comparisons test :Pla2g7* | *Mean Diff.* | | *95% CI of diff.* | | | *Significant?* | | | *Summary* | | |  |
|  |  | |  | | |  | | |  | | |  |
| *E14.25 vs. E15.25* | *7.641* | | *-67.56 to 82.84* | | | *No* | | | *ns* | | |  |
| *E14.25 vs. E17.25* | *-7.541* | | *-82.74 to 67.66* | | | *No* | | | *ns* | | |  |
| *E14.25 vs. E20* | *-134.7* | | *-209.9 to -59.45* | | | *Yes* | | | ***** | | |  |
| *E15.25 vs. E17.25* | *-15.18* | | *-90.39 to 60.02* | | | *No* | | | *ns* | | |  |
| *E15.25 vs. E20* | *-142.3* | | *-217.5 to -67.09* | | | *Yes* | | | ***** | | |  |
| *E17.25 vs. E20* | *-127.1* | | *-202.3 to -51.91* | | | *Yes* | | | ***** | | |  |
| *Tukey's multiple comparisons test :Pla2g1b* | *Mean Diff.* | | *95% CI of diff.* | | | | *Significant?* | | | *Summary* | | |
|  |  | |  | | | |  | | |  | | |
| *E14.25 vs. E15.25* | *4.197* | | *-9.747 to 18.14* | | | | *No* | | | *ns* | | |
| *E14.25 vs. E17.25* | *5.874* | | *-8.071 to 19.82* | | | | *No* | | | *ns* | | |
| *E14.25 vs. E20* | *-9.658* | | *-23.60 to 4.287* | | | | *No* | | | *ns* | | |
| *E15.25 vs. E17.25* | *1.677* | | *-12.27 to 15.62* | | | | *No* | | | *ns* | | |
| *E15.25 vs. E20* | *-13.85* | | *-27.80 to 0.08962* | | | | *No* | | | *ns* | | |
| *E17.25 vs. E20* | *-15.53* | | *-29.48 to -1.587* | | | | *Yes* | | | *** | | |
| *Tukey's multiple comparisons test :Ptges* | *Mean Diff.* | | *95% CI of diff.* | | | *Significant?* | | | *Summary* | | |  |
|  |  | |  | | |  | | |  | | |  |
| *E14.25 vs. E15.25* | *-17.10* | | *-315.4 to 281.2* | | | *No* | | | *ns* | | |  |
| *E14.25 vs. E17.25* | *-194.4* | | *-492.7 to 103.9* | | | *No* | | | *ns* | | |  |
| *E14.25 vs. E20* | *-847.2* | | *-1145 to -548.9* | | | *Yes* | | | ****** | | |  |
| *E15.25 vs. E17.25* | *-177.3* | | *-475.6 to 121.0* | | | *No* | | | *ns* | | |  |
| *E15.25 vs. E20* | *-830.1* | | *-1128 to -531.8* | | | *Yes* | | | ****** | | |  |
| *E17.25 vs. E20* | *-652.8* | | *-951.1 to -354.5* | | | *Yes* | | | ****** | | |  |
| *Tukey's multiple comparisons test :Tbxas1* | *Mean Diff.* | | *95% CI of diff.* | | | *Significant?* | | | *Summary* | | |  |
|  |  | |  | | |  | | |  | | |  |
| *E14.25 vs. E15.25* | *3.172* | | *-16.93 to 23.27* | | | *No* | | | *ns* | | |  |
| *E14.25 vs. E17.25* | *4.283* | | *-15.82 to 24.38* | | | *No* | | | *ns* | | |  |
| *E14.25 vs. E20* | *-36.77* | | *-56.87 to -16.66* | | | *Yes* | | | ***** | | |  |
| *E15.25 vs. E17.25* | *1.111* | | *-18.99 to 21.21* | | | *No* | | | *ns* | | |  |
| *E15.25 vs. E20* | *-39.94* | | *-60.04 to -19.84* | | | *Yes* | | | ***** | | |  |
| *E17.25 vs. E20* | *-41.05* | | *-61.15 to -20.95* | | | *Yes* | | | ****** | | |  |
| *Tukey's multiple comparisons test :Akr1b1* | | *Mean Diff.* | | *95% CI of diff.* | *Significant?* | | | *Summary* | | |  |  |
|  | |  | |  |  | | |  | | |  |  |
| *E14.25 vs. E15.25* | | *1179* | | *-90.49 to 2448* | *No* | | | *ns* | | |  |  |
| *E14.25 vs. E17.25* | | *1542* | | *272.7 to 2812* | *Yes* | | | *** | | |  |  |
| *E14.25 vs. E20* | | *-1416* | | *-2685 to -146.1* | *Yes* | | | *** | | |  |  |
| *E15.25 vs. E17.25* | | *363.2* | | *-906.3 to 1633* | *No* | | | *ns* | | |  |  |
| *E15.25 vs. E20* | | *-2595* | | *-3864 to -1325* | *Yes* | | | ****** | | |  |  |
| *E17.25 vs. E20* | | *-2958* | | *-4227 to -1688* | *Yes* | | | ****** | | |  |  |
| *Tukey's multiple comparisons test :Ptgis* | | *Mean Diff.* | | *95% CI of diff.* | *Significant?* | | | *Summary* | | |  |  |
|  | |  | |  |  | | |  | | |  |  |
| *E14.25 vs. E15.25* | | *264.9* | | *-167.4 to 697.2* | *No* | | | *ns* | | |  |  |
| *E14.25 vs. E17.25* | | *1124* | | *691.5 to 1556* | *Yes* | | | ****** | | |  |  |
| *E14.25 vs. E20* | | *1120* | | *687.4 to 1552* | *Yes* | | | ****** | | |  |  |
| *E15.25 vs. E17.25* | | *858.9* | | *426.6 to 1291* | *Yes* | | | ***** | | |  |  |
| *E15.25 vs. E20* | | *854.8* | | *422.5 to 1287* | *Yes* | | | ***** | | |  |  |
| *E17.25 vs. E20* | | *-4.094* | | *-436.4 to 428.2* | *No* | | | *ns* | | |  |  |

**Supplemental Figure S1**. **A)** Differential expression of other genes related to the endocannabinoid pathway *Pla1a, Pla2g2a, Pla2g4a, Pla2g7, Pla2g1b* (Phospholipase A group, involved upstream in synthesis of Arachidonic acid) and *Ptges, Tbxas1, Akr1b1, Ptgis* (Downstream genes involved in metabolism of PGH2 derivatives) screened in Microarray (RatRef-12 arrays Illumina). X- Axes depict Gestational Ages E14.25, E15.25, E17.25 and E20. Y-Axes are the Microarray Hybridization Signals. **B)** Statistical Analyses by One way ANOVA –Multiple Comparisons between all four gestational age groups E14.25, E15.25, E17.25 and E20. Significance was ascribed where gene expression was changed by < 2 (∗

𝑃 < 0.05;∗∗ 𝑃 < 0.005; ∗∗∗ 𝑃 < 0.001; ∗∗∗∗ 𝑃 < 0.0001; and ns 𝑃 > 0.05).


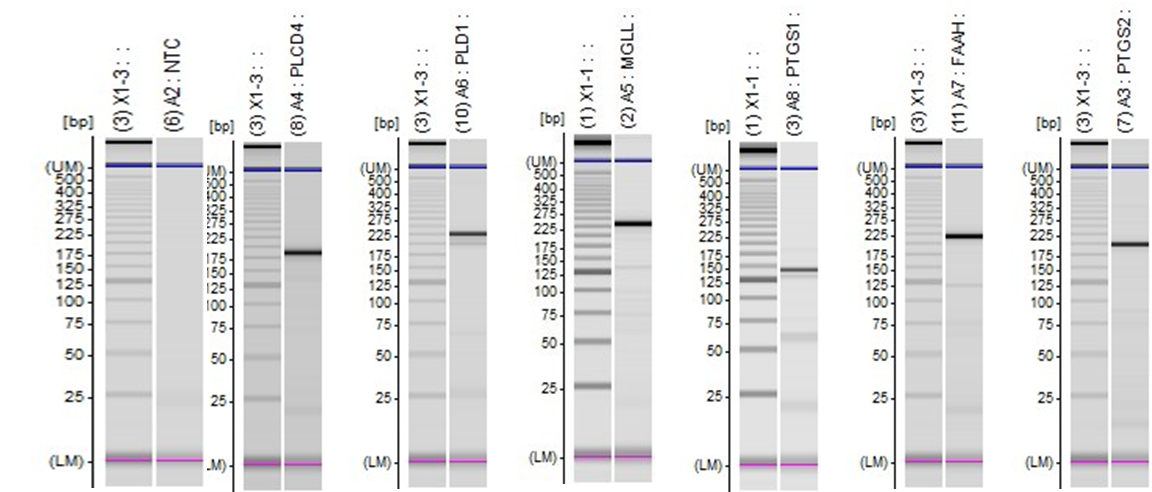


**Supplemental Figure S2:** Primer testing on PCR prior to qPCR experiments using Shimadzu MULTINA™.
